# Fabrication and Characterization of Docosahexaenoic Acid Algal Oil Pickering Emulsions Stabilized Using the Whey Protein Isolate–High-Methoxyl Pectin Complex

**DOI:** 10.3390/foods13132159

**Published:** 2024-07-08

**Authors:** Zhe Yu, Li Zhou, Zhe Chen, Ling Chen, Kunqiang Hong, Dongping He, Fenfen Lei

**Affiliations:** 1 College of Food Science and Engineering, Wuhan Polytechnic University, Wuhan 430023, China; yz13454484414@163.com (Z.Y.); liz358@usask.ca (L.Z.); cl1413928750@163.com (L.C.); hongkq@whpu.edu.cn (K.H.); hedp123456@163.com (D.H.); 2 Key Laboratory of Edible Oil Quality and Safety for State Market Regulation, Wuhan 430023, China; whpuchenzhe@163.com; 3 Wuhan Institute for Food and Cosmetic Control, Wuhan 430023, China; 4 Key Laboratory for Deep Processing of Major Grain and Oil, Ministry of Education, Wuhan 430023, China

**Keywords:** DHA, Pickering emulsion, stability, whey protein isolate, high-methoxyl pectin

## Abstract

In this study, the whey protein isolate–high-methoxyl pectin (WPI-HMP) complex prepared by electrostatic interaction was utilized as an emulsifier in the preparation of docosahexaenoic acid (DHA) algal oils in order to improve their physicochemical properties and oxidation stability. The results showed that the emulsions stabilized using the WPI-HMP complex across varying oil-phase volume fractions (30–70%) exhibited consistent particle size and enhanced stability compared to emulsions stabilized solely using WPI or HMP at different ionic concentrations and heating temperatures. Furthermore, DHA algal oil emulsions stabilized using the WPI-HMP complex also showed superior storage stability, as they exhibited no discernible emulsification or oil droplet overflow and the particle size variation remained relatively minor throughout the storage at 25 °C for 30 days. The accelerated oxidation of the emulsions was assessed by measuring the rate of DHA loss, lipid hydroperoxide levels, and malondialdehyde levels. Emulsions stabilized using the WPI-HMP complex exhibited a lower rate of DHA loss and reduced levels of lipid hydroperoxides and malondialdehyde. This indicated that WPI-HMP-stabilized Pickering emulsions exhibit a greater rate of DHA retention. The excellent stability of these emulsions could prove valuable in food processing for DHA nutritional enhancement.

## 1. Introduction

Docosahexaenoic acid (DHA), an essential ω-3 polyunsaturated fatty acid, plays a critical role in the proliferation, migration, differentiation, and synapsis of nerve cells within the brain. Additionally, it prevents cardiovascular diseases and reduces the risk of coronary heart disease, Alzheimer’s disease, and myocardial infarction [1,2]. The correlation between adequate DHA intake and the maintenance of normal physiological functions in the human body is well established. However, given the human body’s inability to synthesize DHA due to a lack of endogenous enzymes, external supplementation is necessary [3]. However, DHA is characterized by high hydrophobicity and oxidative sensitivity, making it challenging to directly incorporate DHA into water-based food matrices, thus significantly limiting the utilization of DHA [4,5]. Therefore, improving the physical and oxidative stability of DHA algal oil and enhancing its solubility are paramount concerns in the development of functional foods containing DHA algal oil.

In the food industry, emulsions have found application in the delivery of bioactive compounds. Due to their advantages, including environmental friendliness, low toxicity, and good stability, Pickering emulsions have found wide applications in the food, pharmaceutical, and cosmetic sectors. The solid particles in Pickering emulsions are adsorbed at the oil–water interface, thereby enhancing the stability of the oil–water interface layer [6]. Compared with traditional emulsions, oil-in-water emulsions are stabilized by mitigating surface tension and reducing surfactant usage [7,8]. Various protein–polysaccharide nanoparticles have been utilized for stabilizing Pickering emulsions intended for DHA delivery. Soy protein isolate–chitosan nanoparticles have been employed to stabilize DHA Pickering emulsions, followed by investigations into their in vitro digestion and toxicity [9]. Yan et al. [10] employed octenyl succinic anhydride-modified chitosan as a barrier to enhance the stability of DHA-loaded nano-emulsions. Additionally, alternative materials such as zein-poly (lactic-co-glycolic acid) (PLGA) and chitosan have been used for DHA delivery systems [11]. While studies have primarily focused on the preparation, stability, and digestion characteristics of DHA Pickering emulsions, further exploration into the influence of Pickering emulsions on the oxidative stability of DHA is warranted.

Protein, as an amphiphilic biopolymer, possesses good interfacial adsorption and emulsifying properties alongside high nutritional value, rendering it an optimal choice for stabilizing food-grade Pickering emulsions [12]. Whey protein isolate (WPI) exhibits high nutritional value and good functional characteristics, such as emulsification, foaming, solubility, and ligand-binding properties. These characteristics contribute to improving the texture, sensory attributes, and processing conditions of food; thus, WPI finds extensive utilization in food research [13,14]. Pectin, a polysaccharide extracted from fruits, has been traditionally used as a gelling agent in the food industry due to its good physicochemical properties, film-forming properties, and widespread availability. High-methoxyl pectin (HMP) displays excellent bonding capabilities with protein, even under high-temperature conditions. Protein and pectin can aggregate through electrostatic interactions, hydrophobic interactions, hydrogen bonding, and steric hindrance, thereby improving the stability of the emulsion [15]. Combining protein and polysaccharide yields composite particles with good two-phase wettability, suitable for stabilizing Pickering emulsions. Compared with singular protein or polysaccharide particles, composite particles, such as Pickering stabilizers, exhibit superior emulsifying performance [16,17]. The utilization of these composite particles not only circumvents the need for synthetic modifiers in chemical modification, but also imparts greater stability to Pickering emulsions. Some researchers have found that employing WPI-HMP composite particles for lipid embedding in Pickering emulsions effectively retards lipid oxidation and preserves nutrients efficiently [18]. Incorporating curcumin into WPI-HMP emulsions has been shown to enhance encapsulation properties and antioxidant activity [15]. However, the application of the WPI-HMP complex for delivering DHA algal oil via the Pickering emulsion system remains unexplored.

In this study, the WPI-HMP complex formed through electrostatic interaction was employed to produce DHA algal oil Pickering emulsion in order to improve the physicochemical properties and oxidation stability of DHA algal oil. The ζ-potential, particle size, microstructure, and rheological properties were investigated to evaluate the physical stability of the emulsion. Additionally, the salt stability, thermal stability, and storage stability of the DHA Pickering emulsions were examined to improve the protective and delivery characteristics of the emulsion. Furthermore, oxidative stability was evaluated based on the retention of DHA and malonaldehyde (MDA) levels. By comparative analysis of emulsion stabilized with WPI alone, HMP alone, and the complex of WPI and HMP, this study aimed to develop a DHA algal oil Pickering emulsion with enhanced stability suitable for food processing applications to maximize the nutritional benefits of DHA.

## 2. Materials and Methods

### 2.1. Materials

WPI (purity ≥ 94%) was purchased from Hilmar Ingredients Co., Ltd. (Hilmar, CA, USA), while HMP (esterification degree 78%, biological grade) was obtained from Yantai Anderle Pectin Co., Ltd. (Yantai, China). Nile Red (98% biological grade) was purchased from Shanghai Yuanye Biotechnology Co., Ltd. (Shanghai, China). Docosahexaenoic acid methyl ester (analytical standard ≥ 99%) was purchased from NU-CHEK Co., Ltd. (Elysian, MN, USA). DHA algal oil (DHA content ≥ 40%) was obtained from Hubei Fuxing Biotechnology Co., Ltd. (Wuhan, China). All other chemicals and solvents were of analytical grade and were purchased from Sinopharm Chemical Regent (Shanghai, China).

### 2.2. Preparation of DHA Algal Oil Pickering Emulsion

The WPI and HMP complex ratio was maintained at 1:1, with a total biopolymer concentration of 1% in the solution. The preparation and properties of the WPI-HMP complex could refer to our previous study [19]. The pH was adjusted to 3.5 using 0.5 M HCl and 0.5 M NaOH. Subsequently, the mixture was magnetically stirred for 2 h at 300 r min^−1^. The resulting WPI-HMP complex solution was then mixed with DHA algal oil, with oil-phase volume fractions of 30%, 40%, 50%, 60%, and 70% in the emulsion. Following this, the mixture was homogenized at 15,000 rpm for 2 min to obtain HPI-MMP stabilized Pickering emulsions. Emulsions stabilized using either WPI (1%) or HMP (1%) were used as controls, which were prepared in the same way as the WPI-HMP-stabilized emulsions.

### 2.3. Characterization of DHA Algal oil Pickering Emulsion

#### 2.3.1. ζ-potential and Particle Size Distribution

ζ-potential and particle size distribution have important effects on the properties and stability of Pickering emulsion. They were assessed using the Zetasizer Nano ZS90 instrument (Malvern Instruments Ltd., Malvern, UK). The average particle size, polymer dispersity index (PDI), and ζ-potential were measured. The average particle size was denoted as D_4,3_, with its calculation conducted in accordance with Equation (1) and repeated three times for each sample.

(1) $$D_{4,3}=\frac{\sum n_{i}V_{i}d_{i}}{\sum n_{i}V_{i}}$$

In Equation (1), $n_{i}$ represents the number of droplets, while $d_{i}$ represents the diameter of droplets.

#### 2.3.2. Confocal Laser Scanning Microscopy (CLSM)

The microstructure of DHA algal oil Pickering emulsions was investigated by CLSM according to the method outlined by Wang et al. [20]. One ml of emulsion sample was mixed with a total of 40 μL of staining dye (1 mg/mL each of Nile Red and fluorescein isothiocyanate) for 5 min, and then diluted fivefold with distilled water. The stained emulsion was excited with fluorescence excitation at 488 nm and 543 nm, respectively.

#### 2.3.3. Characterization of Rheological Properties

The rheological properties of Pickering emulsions were assessed at 25 °C utilizing a rheometer (Kinnexus Pro+ Rotational, Naichi Instrument Company, Selb, Germany). The tests were performed employing a 60 mm parallel plate geometry with a gap height set at 1.0 mm. Analysis of the storage moduli (G′) and loss moduli (G″) involved frequency and amplitude scans spanning the range of 0.1–100 s^−1^, alongside a strain amplitude of 0.5%. The apparent viscosity was determined through shear sweeps spanning from 0.1 to 100 s^−1^.

### 2.4. Stability of DHA Algal Oil Pickering Emulsions

#### 2.4.1. Salt Stability

Various samples were adjusted using NaCl aqueous solutions to assess changes in the emulsion’s average particle size and ζ-potential. The final concentrations of NaCl in the emulsion were 0, 20, 40, 80, and 160 mmol/L, respectively. Particle sizes and ζ-potential were subsequently determined.

#### 2.4.2. Thermal Stability

Freshly prepared emulsions were transferred into 30 mL penicillin bottles and placed in water bath vessels at 30 °C, 60 °C, and 90 °C for 30 min. Subsequently, they were rapidly cooled in the cold water to room temperature for analysis of particle size and ζ-potential.

#### 2.4.3. Storage Stability

Freshly prepared emulsions were transferred into 30 mL penicillin bottles and stored at 25 °C for 30 days. Subsequently, the creaming index (CI) and alterations in the average particle size of the emulsion over varying storage periods were assessed. The CI was calculated using Equation (2).

(2) $$\mathrm{CI}\%=\frac{H_{s}}{H_{t}}\times 100$$

In Equation (2), $H_{s}$ represents the height of the individual serum layers, while $H_{t}$ represents the height of the entire emulsion.

### 2.5. Oxidative Stability of Emulsions

DHA algal oil samples and emulsions were placed in a constant-temperature incubator set at 40 °C for 14 days. Subsequently, appropriate samples were extracted at predetermined intervals for the assessment of relevant indices to evaluate the oxidative stability of DHA algal oil in the emulsion. Prior to the determination of these indices, the oil phase was separated via high-speed centrifugation, facilitating a comparative analysis with non-emulsified algal oil [21].

#### 2.5.1. Determination of DHA Content

DHA content was analyzed by GC-MS (450-GC/240-MS, Varian, Santa Clara, CA, USA) following the method described by Zhang et al. [22]. Initially, a 0.2 g sample was mixed with 2 mL of KOH-CH_3_OH solution (0.5 mol L^−1^) and kept in a water bath at 60 °C for 30 min. Then, 2 mL of Boron trifluoride-methanol was added to the solution and incubated at 60 °C for 3 min. Following this, 2 mL of saturated NaCl solution and 2 mL of n-hexane were added, and thoroughly mixed. The supernatant was then extracted, filtered, and injected into the injection bottle for analysis. An HP-88 (100 m × 0.25 mm × 0.2 μm) chromatographic column was used for separation. Split injection was performed at a ratio of 1:100 taking high-purity nitrogen as carrier gas, with 1 µL injection volume. The inlet temperature was 270 °C. For temperature programming, the oven was maintained at 100 °C for 13 min, increased at intervals of 10 °C min^−1^ to 180 °C and kept there for 6 min, then raised at a rate of 1 °C min^−1^ to 200 °C and kept there for 20 min, and further raised to 230 °C at a rate of 4 °C min^−1^ and balanced for 10 min. Quantitative analysis of DHA was performed using Equation (3).

(3) $$X=\frac{A_{DHA}\times m_{S_{DHA}}\times F_{TG_{DHA-FA_{DHA}}}}{A_{S_{DHA}}\times m}\times 100$$

In Equation (3), X denotes the content of DHA in the sample to be tested, with the unit expressed as grams per hundred grams (g/100 g). $A_{DHA}$ denotes the peak area of methyl DHA in the sample to be tested. $m_{S_{DHA}}$ represents the mass of DHA triglyceride contained in the standard measuring solution (mg). $F_{TG_{DHA}-FA_{DHA}}$ stands for the conversion coefficient of DHA triglyceride into fatty acid, which is 0.9628.

#### 2.5.2. Determination of Hydrogen Peroxide Content

The hydrogen peroxide levels were determined according to the method outlined by Wang et al. [20]. A 0.3 mL aliquot of the emulsion was mixed with 1.5 mL of an iso-octane/isopropanol mixture (3:1, *v*/*v*) and vortexed three times. Subsequently, the mixture was centrifuged at 2000× *g* rpm for 5 min at 4 °C, after which 200 μL of the upper organic phase was transferred to a 5 mL Eppendorf tube. To this, a solution consisting of methanol and 1-butanol in a ratio of 2:1 (*v*/*v*), totaling 2.8 mL, was added. Subsequently, 15 μL of 3.94 mol/L ammonium thiocyanate and 15 µL of ferrous iron solution were added, and the resultant mixture was incubated for 20 min in the dark. The absorbance of the solution was subsequently measured at 510 nm. Hydroperoxide concentrations were deduced from a standard curve of hydrogen peroxide.

#### 2.5.3. Determination of Malondialdehyde (MDA) Content

Thiobarbituric acid reactive substance (TBARS) determination, serving as an indicator of the development of lipid oxidation products, was conducted to assess oxidative stability in the emulsions. The mixture was placed in a thermostatic oscillator set at 50 °C and shaken for 30 min. Subsequently, it was filtered through double-layer quantitative slow filter paper, with the initial filtrate discarded and the remaining filtrate reserved for later use. Following this, 5 mL of the filtrate, along with a standard series of sample solutions, was added to a 25 mL colorimetric tube. Additionally, another 5 mL of a trichloroacetic acid mixture was vortexed with 5 mL of a thiobarbituric acid (TBA) aqueous solution to serve as a sample blank. The resulting mixture was then heated at 90 °C for 30 min in a water bath. After cooling to room temperature, TBARS values were measured using a spectrophotometer at 532 nm. Quantification was performed using a standard curve prepared using 1,1,3,3-tetraethoxypropane. The TBARS values were expressed as the content of MDA (mg kg^−1^).

### 2.6. Statistical Analysis

All samples were prepared in triplicate, and the results were expressed as the mean ± standard deviation. The differences between groups were analyzed using one-way analysis of variance (ANOVA) and Duncan post hoc tests using the Statistical Package for the Social Sciences (SPSS) version 27.0 (IBM Corp., Armonk, NY, USA), with different letters indicating significant differences (*p* < 0.05).

## 3. Results and Discussion

### 3.1. Characterization of DHA Algal Oil Pickering Emulsion

#### 3.1.1. Particle Size Distribution and Average Particle Size

**Figure 1:**
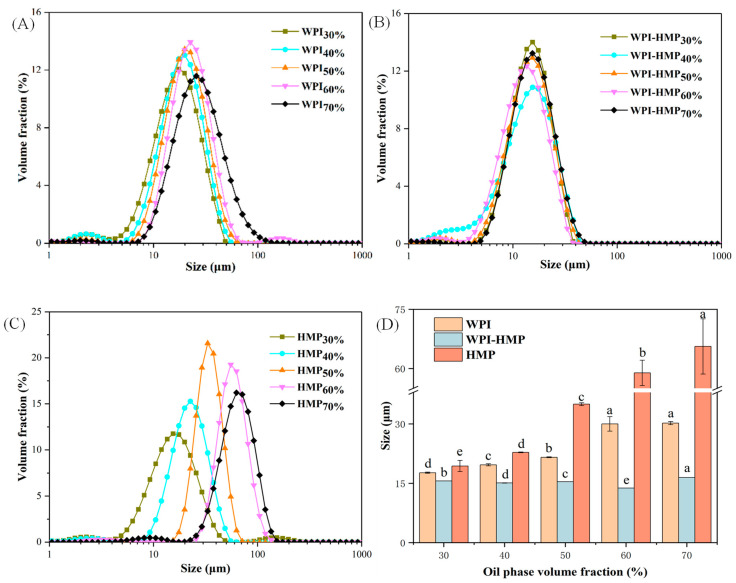
Particle size distribution of WPI- (**A**), WPI-HMP- (**B**), and HMP- (**C**) stabilized emulsions with different oil-phase volume fractions, and the average particle size (D_4,3_) of emulsion with different oil-phase volume fractions (**D**).

The particle size distribution and average particle-size changes of various DHA algal oil Pickering emulsions are shown in Figure 1 (oil phase 30%, 40%, 50%, 60%, and 70%). All emulsions exhibited a unimodal distribution, indicating a relatively uniform distribution across emulsions. Figure 1B,D depict the fact that, with the increase in the volume fraction of the oil phase, the droplet distribution of WPI-HMP complex-stabilized Pickering emulsion remained unchanged, and the average particle size exhibited no discernible increase. Conversely, as the volume fraction of the oil phase increased, the particle size distribution and the average particle size (D_4,3_) of emulsions stabilized solely using WPI or HMP increased significantly and shifted towards larger values, accompanied by a significant increase in average particle size (D_4,3_). This suggests that the introduction of HMP into the WPI solution increased the viscosity of the water phase, fostering a network structure that binds oil droplets and diminishes droplet aggregation and the emulsion’s particle size. As the volume fraction of the oil phase increased, the most pronounced increase in average particle size was observed in the emulsion stabilized by HMP, with HMP demonstrating relatively low emulsifying activity and emulsifying stability. Excess oil phase results in inadequate coverage of oil droplets by pectin, and the repulsion between droplets decreases [23], which is insufficient to inhibit the aggregation of droplets, potentially leading to partial emulsion flocculation. These results demonstrate that the formation of a complex between WPI and HMP via electrostatic interactions enhances the stability of middle and high internal-phase Pickering emulsions.

#### 3.1.2. Microstructure of the Various DHA Algal Oil Pickering Emulsions

**Figure 2:**
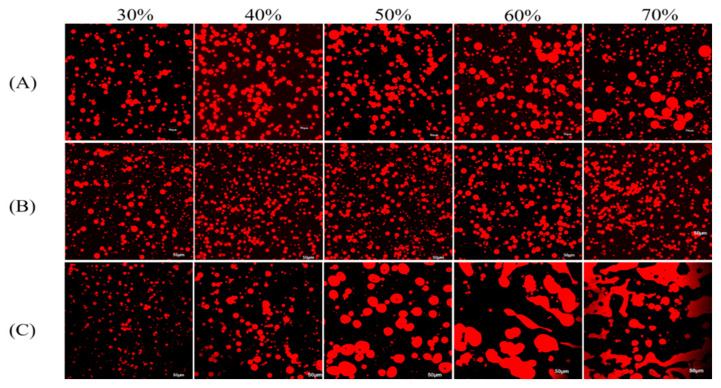
Laser confocal plots of WPI- (**A**), WPIHMP-(**B**), and HMP (**C**) stabilized emulsions; DHA oil was previously stained by Nile red. The oil-phase volume fractions are 30%, 40%, 50%, 60%, and 70%, from left to right.

The morphology of DHA algal oil Pickering emulsions stabilized using WPI, WPI-HMP, and HMP is shown in Figure 2. As illustrated in Figure 2A, the emulsion stabilized using WPI exhibited a smaller particle size and a more uniform distribution within the 30–50% oil-phase volume fraction range. With an increase in the volume fraction of the oil phase, larger droplets became apparent in the emulsion. This phenomenon became progressively obvious as the oil-phase volume fraction increased, resulting in a gradual deterioration in the uniformity of droplet dispersion. Generally, emulsion stability improved as droplet size decreased, provided that the WPI emulsifier content was sufficient. This was attributed to the larger specific surface area of tiny droplets, facilitating more comprehensive WPI adsorption at the oil–water interface and the consequential vital repulsion between droplets. Hence, the droplets were less prone to aggregation and exhibited good dispersibility. However, when the available quantity of WPI was fixed, an increase in the oil-phase volume fraction led to insufficient coverage of oil droplets by WPI. Consequently, oil droplets could only be covered by enlarging the particle size and reducing the specific surface area [24], a finding consistent with that reported by Yang [25]. As shown in Figure 2C, at a volume fraction of 50% for the oil phase, the droplet morphology of the HMP-stabilized emulsion exhibited deformation, and it no longer displayed regular spherical droplets. With further increments in the oil phase volume, the red signal became dispersed in sheets, indicating a lack of HMP adsorption on the surface of most oil droplets. This observation is consistent with the results of the particle size measurements outlined in Section 3.1.1. The middle and high internal-phase emulsions stabilized using WPI or HMP alone tended to aggregate easily and lose stability. The droplet size of the WPI-HMP-stabilized Pickering emulsion remained unaffected by variations in the oil phase volume. As shown in Figure 2B, upon the addition of HMP, the WPI-stabilized emulsion exhibited spherical droplets characterized by small particle sizes across the oil-phase volume fraction range of 30% to 70%, which were more uniformly distributed in the continuous phase. This can be attributed to the formation of a dense network structure by HMP in the continuous phase, wherein the electrostatic assembly binds the oil droplets, thereby inhibiting droplet aggregation and improving the stability of Pickering emulsions.

#### 3.1.3. Analysis of Rheological Behavior

**Figure 3:**
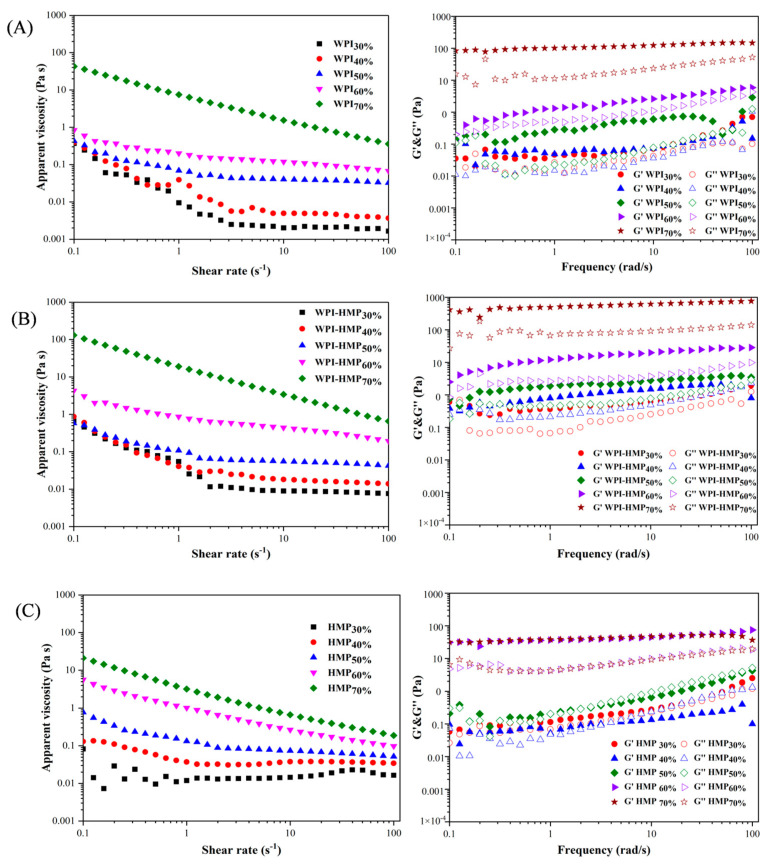
Apparent viscosity and storage-energy modulus of WPI- (**A**), WPI-HMP- (**B**), and HMP- (**C**) stabilized emulsions with different oil-phase volume fractions (30−70%).

As shown in Figure 3, the viscosity of all emulsions gradually decreased with increasing shear rate, indicative of typical shear-thinning behavior, thereby signifying the non-Newtonian nature of all emulsions [26]. As the volume fraction of the oil phase increased, the initial apparent viscosities of the three emulsions increased notably. This occurs due to the high-volume fraction of the oil phase, resulting in closely packed droplets. Consequently, the interaction among oil droplets improves the system’s viscosity. This serves as the primary factor contributing to improved stability of the emulsion. Simultaneously, we found that emulsion stabilized using WPI-HMP demonstrated higher viscosity compared to other emulsions with identical volume fractions of the oil phase. This observation underscores the thickening effect exerted by HMP on the emulsion system. Therefore, the high viscosity of the WPI-HMP complex could enhance both the storage stability of the emulsion and the texture of food.

As the volume fraction of the oil phase increased, the G′ and G″ of emulsions stabilized by the three emulsifiers exhibited a rising trend. This may be attributed to the increasing volume fraction of the oil phase, resulting in more densely packed droplets, which was more conducive to the formation of gel-like structures [23]. For emulsions stabilized using WPI and WPI-HMP, the G′ consistently surpassed the G″ across the entire frequency range, indicating the ability of these emulsions to form gel-like structures with robust elasticity across varying oil-phase volume fractions [27]. In addition, compared to emulsions stabilized by other emulsifiers, the G′ and G″ of WPI-HMP-stabilized Pickering emulsion were higher at any given oil-phase volume fraction. This further demonstrates that HMP improves the deformation resistance and viscoelasticity of emulsions [28]. The G′ and G″ of the emulsion stabilized using HMP alone exhibited notable changes at various oil-phase volume fractions. When the oil-phase volume fraction was below 50%, G′ and G″ exhibited a crossing crossover phenomenon with an increase in oscillation frequency. This can be attributed to the disruption of the gel-like structure of the emulsion induced by higher oscillation frequencies. Consequently, oil droplets permeated the emulsion and underwent re-arrangement. When the volume fraction of the oil phase reached 60%, G′ was greater than G″ and no crossover was observed, indicating that the increase in oil-phase volume fraction increased the bulk density of emulsion droplets and consequently improved the viscoelasticity of the emulsion.

### 3.2. Stability of Different DHA Algal Oil Pickering Emulsions

#### 3.2.1. Salt Stability

**Figure 4:**
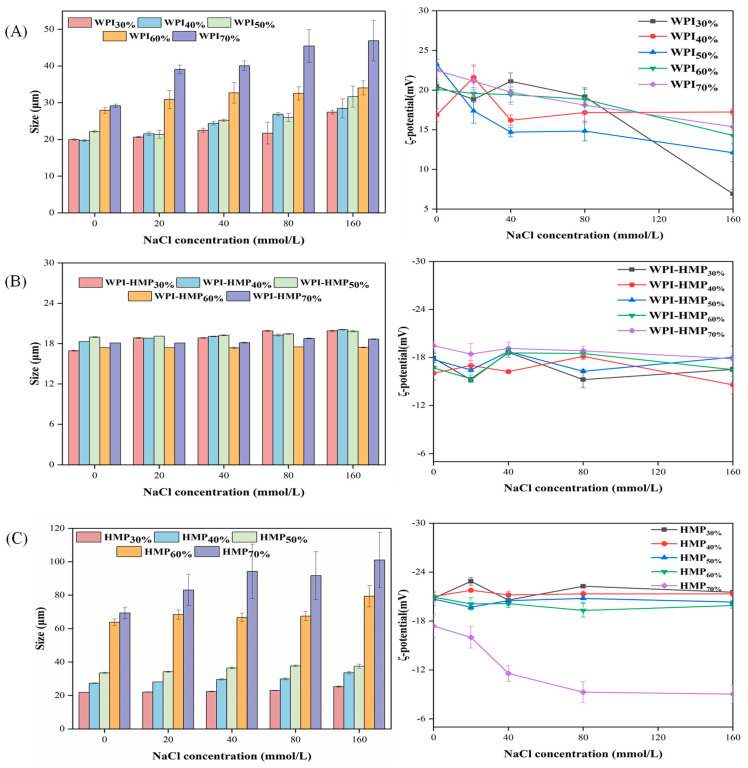
Changes in particle size and ζ-potential of emulsions stabilized by WPI (**A**), WPI−HMP (**B**), and HMP (**C**) with different oil-phase volume fractions (30−70%) at different salt ion concentrations.

The particle size and ζ-potential of emulsions at various NaCl concentrations were measured, with the results shown in Figure 4. Salt ions exerted minimal effect on WPI-HMP-stabilized emulsions across the examined concentration range. As the ion concentration increased, there was no significant increase observed in the particle size of the emulsion, irrespective of the differing oil-phase volume fractions. This phenomenon can be attributed to the formation of a negatively charged protective layer by HMP and WPI, effectively impeding the aggregation of emulsion droplets [29]. However, the stability of emulsions stabilized solely using WPI or HMP was significantly affected by salt ion concentration. As shown in Figure 4A, the particle size of WPI-stabilized emulsions tended to increase with both increasing salt ion concentration and increasing volume fraction of the oil phase. Moreover, the salt ion concentration exerted a more significant influence on the emulsion. This is likely due to the electrostatic shielding effect of salt ions [30], which reduces electrostatic repulsion between droplets, thereby promoting aggregation and consequent enlargement of particle size. As depicted in Figure 4C, in HMP-stabilized emulsions with oil-phase volume fractions below 60%, the particle size remained relatively unchanged. This can be rationalized by the abundance of negative charges on the emulsion surface at lower oil-phase volume fractions, fostering robust repulsion between droplets and impeding droplet aggregation [29]. However, as the oil-phase volume fraction reached 70%, the emulsifying efficacy of HMP became limited, as its capacity to envelop oil droplets was insufficient, leading to easier aggregation and merger and thereby increasing particle size.

Regarding ζ-potential, the WPI-stabilized emulsions demonstrated a decreasing trend with increasing NaCl concentration, attributable to the electrostatic shielding effect exerted by salt ions. In contrast, the ζ-potential of the WPI-HMP composite-stabilized emulsions displayed less susceptibility to variations in NaCl concentration. This indicates that the electrostatic interaction between WPI and HMP engenders a more robust spatial separation among emulsion droplets. Additionally, a higher oil content in the emulsion increases its viscosity, thereby retarding the rate of droplet aggregation and merger. For HMP-stabilized emulsions with low oil-phase volume fractions, the NaCl concentration exerted negligible impact on the ζ-potential, albeit HMP’s emulsifying capability was limited. These results suggest that emulsions featuring high oil-phase volume fractions exhibit lower ion stability.

#### 3.2.2. Thermal Stability

**Figure 5:**
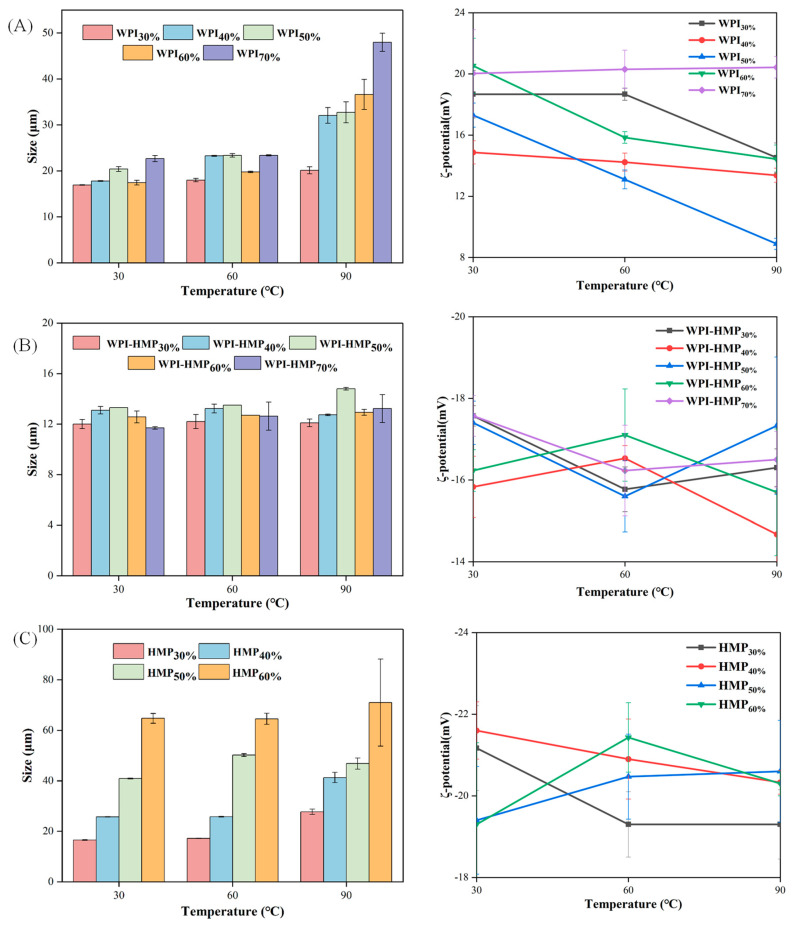
Changes in particle size and ζ-potential of emulsions stabilized by WPI (**A**), WPI−HMP (**B**), and HMP (**C**) with different oil-phase volume fractions after treatment for 30 min at different temperatures.

Thermal sterilization is an essential process in the food industry. Investigating the impact of heat treatment on the stability of Pickering emulsions holds significant practical importance. Figure 5 illustrates variations in the average particle size and ζ-potential of WPI-, WPI-HMP-, and HMP-stabilized emulsions across differing oil-phase volume fractions, subsequent to 30 min of heating at various temperatures. Based on visual appearance the HMP-stabilized emulsion containing a 70% oil-phase volume fraction was separated into water and oil layers during heating, so it is not shown in Figure 5C. As shown in Figure 5B, compared with the emulsions stabilized solely using WPI or HMP, the particle size of the WPI-HMP-stabilized Pickering emulsion exhibited no obvious alteration following heat treatment across varying temperatures, indicating that the emulsion stabilized by WPI-HMP displayed superior thermal stability. This may be attributed to the heightened gelation degree of the WPI-HMP-stabilized emulsion following exposure to elevated temperatures. As the heating temperature increased, the particle size of WPI-stabilized emulsion displayed a continuous increase, likely caused by the denaturation of WPI at high temperatures, the exposure of hydrophobic groups embedded within protein, the attraction between droplets through hydrophobic interactions, and the aggregation of droplets [31]. Upon further elevation of temperature to 90 °C, the particle size of the HMP-stabilized emulsion notably enlarged. It was evident that the HMP-stabilized emulsion was sensitive to heat, potentially linked to the inherent thermal stability of pectin. Studies have shown that heat treatment of pectin under acidic conditions reduces its viscosity and disrupts the original three-dimensional network structure of pectin, thereby diminishing the binding ability of the network structure to the oil droplets, promoting droplet aggregation, and consequently reducing the stability of the emulsion [32].

The ζ-potential of the different emulsions after being heated at various temperatures is illustrated in Figure 5. Generally, the absolute value of the ζ-potential of emulsions exhibited a declining trend with increasing heat-treatment temperature. Additionally, the emulsion with stable WPI demonstrated the most pronounced change. This can be attributed to the denaturation of WPI induced by heat treatment, leading to the disruption of its adsorption arrangement at the oil–water interface. Compared with that of the WPI-HMP-stabilized emulsion, the ζ-potential of the WPI-HMP-stabilized emulsion exhibited minimal alteration, which indicated that WPI-HMP−stabilized emulsions exhibit superior thermal stability.

### 3.3. Storage Stability

The oil−in−water emulsion is a dynamic and unstable delivery system. Over prolonged periods of storage, various alterations occur within the emulsion, such as aggregation, delamination, and demulsification, all of which serve as essential indicators for assessing the stability of emulsions [33]. In this study, the emulsion with a 70% volume fraction of oil phase, stabilized using HMP, exhibited a pronounced demulsification phenomenon. Following storage at room temperature for 72 h, the emulsion separated into oil and water phases. Consequently, emulsion CI and particle size alteration could not be measured, and thus, this part of the data was not reported.

#### 3.3.1. Creaming Index (CI)

**Figure 6:**
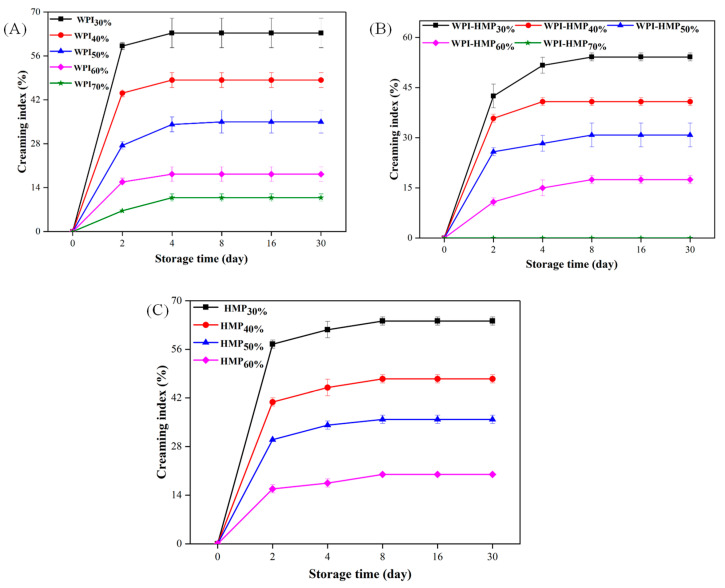
Changes in emulsion index during storage for WPI- (**A**), WPI-HMP-(**B**), and HMP- (**C**) stabilized emulsions with different oil-phase volume fractions.

The Creaming Index (CI) indicates the delamination degree of emulsions and offers a means to evaluate the stability of the emulsion. As shown in Figure 6, the CI exhibited a rapid initial increase followed by a more gradual incline. At equivalent oil-phase volume fractions, the WPI-HMP-stabilized Pickering emulsion showed a lesser degree of stratification compared to emulsions stabilized solely using WPI or HMP. This indicated that the WPI-HMP complex is more effective in preserving the structure of the emulsion. In addition, at constant concentrations of WPI, WPI-HMP, and HMP, the CI tended to decrease with increasing oil-phase volume fractions. At an oil-phase volume fraction of 70%, the WPI-HMP-stabilized Pickering emulsion exhibited no discernible demulsification or oil droplet overflow during a 30-day storage period, indicative of good stability. This can be attributed to the enhanced viscoelasticity of the emulsion resulting from the increased oil-phase volume fraction, leading to a closely arranged droplet configuration resembling a gel-like structure. The formation of such a gel-like emulsion prevented the flow and aggregation of oil droplets, thereby improving the stability of the emulsion. Similar results have been reported for WPI–phytosterol-stabilized Pickering emulsions [34]. 

#### 3.3.2. Particle Size Analysis

**Figure 7:**
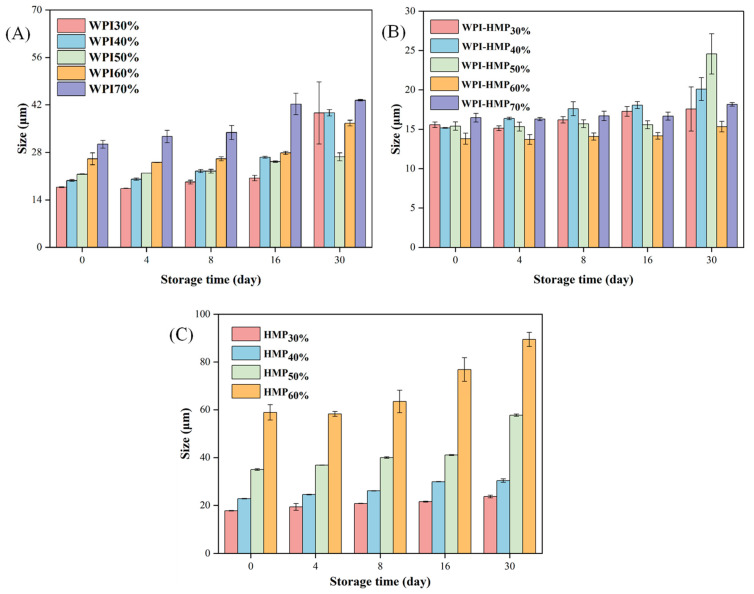
Mean particle size variation of WPI- (**A**), WPI−HMP- (**B**), and HMP- (**C**) stabilized emulsions with different oil−phase volume fractions during storage.

As depicted in Figure 7A, the average particle size of the WPI-stabilized emulsion gradually increased over the 30-day storage period. There was a pronounced increase in the particle size of the emulsion from the 16th to the 30th day, indicating substantial droplet aggregation during the middle and later stages of storage, which increased emulsion particle size. It was evident that the storage stability of emulsions stabilized using WPI was poor. As shown in Figure 7B, the particle size variation of the WPI-HMP composite-stabilized emulsion remained relatively minor throughout the entire storage duration, indicating the superior storage stability of WPI-HMP composite-stabilized emulsions. This was because WPI and HMP formed a barrier through electrostatic interactions, which improved the emulsifying stability and adsorption capacity of WPI at the oil-water interface, thereby preventing the aggregation of droplets and effectively improving the storage stability of the emulsion. Moreover, the particle size of HMP-stabilized emulsions with 30%-40% oil-phase volume fraction did not exhibit significant alterations during storage, owing to a robust net negative charge on the droplet surface, which fostered electrostatic repulsion between droplets, inhibiting aggregation. However, with a continued increase in oil content, the low coverage of HMP on the droplet surface could not provide an effective barrier for protecting the droplets [35], thereby promoting droplet aggregation and subsequent particle size enlargement.

### 3.4. Oxidation Stability of Various DHA Algal Oil Pickering Emulsions

The shelf life of emulsion products is contingent upon lipid oxidation. In this study, the oxidative stability of DHA algal oil within the emulsion was examined by monitoring the changes in DHA content, hydrogen peroxide levels, and MDA content during accelerated oxidation.

#### 3.4.1. Changes in DHA Content during Accelerated Oxidation

**Figure 8:**
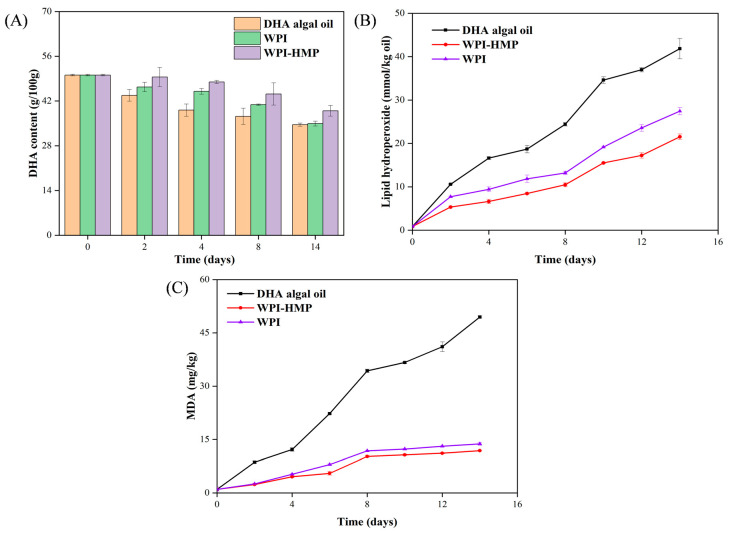
Oxidative stability analysis of different emulsion systems. (**A**) Plots of the changes in DHA content in WPI−, WPI−HMP−stabilized algal oil emulsions and DHAalgal oil during accelerated oxidation; (**B**) changes in hydrogen peroxide values in WPI-, WPI-HMP-stabilized algal oil emulsions and DHA algal oil; (**C**) changes in malondialdehyde content in WPI-, WPI-HMP-stabilized algal oil emulsions and DHA algal oil.

As shown in Figure 8A, as the accelerated oxidation duration increased, the DHA content in all samples exhibited a declining trajectory. Following a 14−day storage period, the DHA content in DHA algal oil and WPI- and WPI-HMP-complex-stabilized emulsion decreased by 30.91%, 30.26%, and 22.28%, respectively. These results indicate that the reduction in DHA content was least pronounced in the WPI-HMP−stabilized Pickering emulsion. This may be because the interaction between HMP and WPI improved the emulsifying efficacy and the interfacial adsorption capacity of WPI, thereby forming a dense physical barrier at the oil–water interface and effectively preventing contact between oxygen molecules and oil droplets. Therefore, it is feasible to use the WPI−HMP complex for the stabilization of Pickering emulsion as an encapsulated delivery system for DHA.

#### 3.4.2. Changes in Hydrogen Peroxide Levels during Accelerated Oxidation

The primary oxidation degree of DHA algal oil emulsions and DHA algal oil was evaluated based on the hydroperoxide content produced. As shown in Figure 8B, the hydrogen peroxide levels of all samples gradually increased with an increase in the accelerated oxidation duration. Specifically, the rate of increase in hydrogen peroxide levels was most pronounced in DHA-free algal oil, indicating that the emulsion embedding system attenuates the rate of lipid oxidation. Following 14 days of accelerated oxidation, the hydrogen peroxide level of the WPI-stabilized emulsion was 27.45 mmol/kg oil. In comparison, the WPI-HMP-complex-stabilized emulsion was 21.54 mmol/kg oil at an equivalent concentration. These results demonstrate that the primary oxidation degree of the WPI-HMP-stabilized Pickering emulsion was significantly (*p* < 0.05) lower than that of the WPI-stabilized emulsion. This may be attributed to the formation of an interface network barrier by the WPI-HMP electrostatic complex at the oil–water interface, thereby inhibiting free radical migration and consequently retarding the oil oxidation rate in the emulsion [36]. Previous studies have shown that the utilization of Tween 80 and various proteins for DHA encapsulation significantly improves the oxidative stability of DHA [37].

#### 3.4.3. Changes in MDA Content during Accelerated Oxidation

As shown in Figure 8C, the MDA content in both DHA algal oil and DHA algal-oil emulsions increased with accelerated oxidation duration. The MDA content in emulsion samples exhibited a relatively lower rate of increase compared to that in DHA algal oil. Under the same oxidation durations, the MDA content in WPI-HMP-stabilized emulsions was lower than that in the WPI-stabilized emulsion, thereby affirming the superior oxidation stability of Pickering emulsions stabilized using the WPI-HMP complex. This might be due to the effective barrier formed by the protein and pectin, which improved the oxidative stability of the emulsion [38]. O/W emulsions or gelled emulsions have been fabricated to deliver DHA algal oil under digestive conditions, with MDA levels utilized as indicators to evaluate oxidation stability. The results have shown that oil-in-water emulsions exhibit protective effects against lipid oxidation [35]. These findings were quite important for delaying the oxidation of DHA algal oil and its application in multiple food matrices.

## 4. Conclusions

In this study, DHA algal oil Pickering emulsions stabilized using WPI, HMP, and WPI-HMP were prepared and evaluated. The results showed that the emulsion stabilized using the WPI-HMP complex exhibited a denser interface and a more even distribution of oil droplets with a network structure, which contributed to stability and a more stable elastic gel-like structure. The oil–water volume ratios significantly influenced the stability of DHA algal-oil emulsions. In addition, the WPI-HMP-complex-stabilized Pickering emulsion of DHA algal oil demonstrated enhanced salt ion stability, thermal stability, and storage stability. Furthermore, the oxidation stability of the WPI-HMP-complex-stabilized DHA emulsion was significantly improved compared to DHA-free algal oil. This research lays the foundation for the future utilization of DHA Pickering emulsion in food processing.

## Data Availability

The original contributions presented in the study are included in the article, further inquiries can be directed to the corresponding author.
